# Transactions of the Medical Society of the State of Pennsylvania

**Published:** 1853-11

**Authors:** 


*Transactions of the Medical Society of the State of Pennsylvania. Annual Session held at Philadelphia, May,* 1853.

This annual contains matter of much local interest and value. It would seem, from a cursory perusal, that the society were endeavoring rather to educe facts of general interest to the profession, than to make their Transactions a vehicle for self laudation and mutual admiration. The present volume contains topographical and sanitary reports from different counties, intended to illustrate the peculiar influences of geological formation, altitude, air and water, on the causation of disease, histories of the epidemics of the past year, and topographical maps, which add much to the permanent value of the volume, and serve to make it a production creditable to the society and its members. S. B. H.

